# The dodeca-coordinated La©B_8_C_4_^+/0/−^ molecular wheels: conflicting aromaticity *versus* double aromaticity†

**DOI:** 10.1039/d2ra07155j

**Published:** 2023-01-19

**Authors:** Ying-Jin Wang, Jia-Xin Zhao, Miao Yan, Lin-Yan Feng, Chang-Qing Miao, Cheng-Qi Liu

**Affiliations:** a Department of Chemistry, Xinzhou Teachers University Xinzhou 034000 Shanxi China yingjinwang@sxu.edu.cn

## Abstract

The transition-metal centered boron molecular wheels have attracted the attention of chemists. The highest deca-coordination number for central metal atoms was observed in *D*_10h_ Ta©B_10_^−^ and Nb©B_10_^−^ molecular wheels. Here, we report a theoretical study of La©B_8_C_4_^*q*^ (*q* = +1, 0, −1) clusters with the dodeca-coordinated La atom. The La©B_8_C_4_^*q*^ clusters adopt fascinating molecular wheel structures, showing a La atom enclosed by a perfect B_8_C_4_ monocyclic ring. The cationic La©B_8_C_4_^+^ cluster has a *C*_4v_ symmetry with the distinctly out-of-plane distortion of the La atom (0.70 Å), which is gradually flattened by the sequential reduction reaction. The distortion of the La atom from the plane in the neutral La©B_8_C_4_ cluster decreases to 0.46 Å. The La©B_8_C_4_^−^ species turns out to be perfectly planar. Chemical bonding analyses indicate that the neutral La©B_8_C_4_ and anionic La©B_8_C_4_^−^ possess 10σ and 9π/10π double aromaticity, respectively, obeying the principle of double aromaticity. However, the cationic La©B_8_C_4_^+^ has 10σ and 8π conflicting aromaticity, representing a counterexample in planar hyper-coordinated molecular wheels. The dodeca-coordination number in La©B_8_C_4_^*q*^ (*q* = +1, 0, −1) clusters is unprecedented, which provides a new idea and concept for searching planar hyper-coordinated systems.

## Abstract 2

La©B_8_C_4_^+/0/−^ clusters adopt interesting molecular wheel structures with a dodeca-coordinated La in plane, setting a record for planar structures in chemistry. We provide new ideas and concepts for searching for planar hyper-coordinated systems.

## Graphical abstract

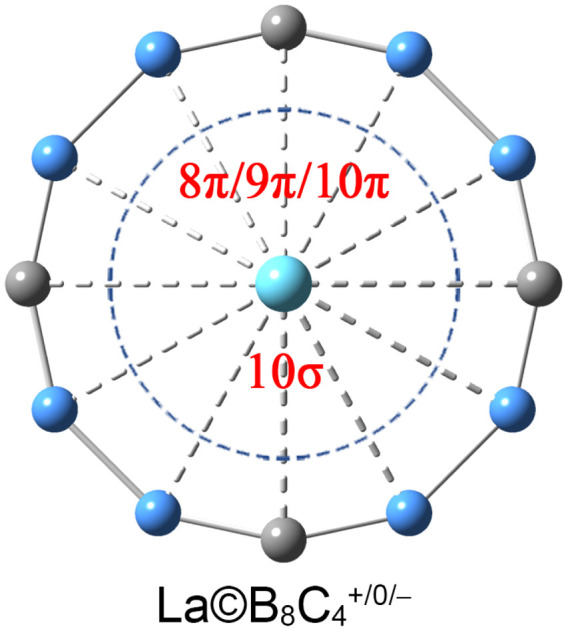

## 1. Introduction

The electron-deficiency of boron results in unconventional geometries in its allotropes and chemical compounds.^1–13^ The bare boron clusters like forming planar or quasi-planar (2D) structures over a wide range of sizes.^4–6,9–17^ The *D*_7h_ B_8_^2−^ and *D*_8h_ B_9_^−^ clusters are intriguing, adopting the perfect molecular wheel shape with a central hepta- and octacoordinate boron atom,^6^ and get their stability from the double (6σ + 6π) aromaticity, fulfilling the 4*N*_σ/π_ + 2 Hückel rule (*N*_σ_ = *N*_π_ = 1). Numerous transition-metal centred boron molecular wheels M©B_*n*_^−^ with the central hypercoordinate metal atom in plane were designed and characterized according to the principle of double aromaticity.^18–21^ At present, the highest coordination number in the planar systems has been limited in Ta©B_10_^−^ and Nb©B_10_^−^.^20,22^ Recently, the metal-centered monocyclic carbon wheel was theoretically investigated with record coordination number of thirteen.^23^ Some main group metal centered boron molecular wheels have also been investigated theoretically.^24,25^

In view of the double aromaticity of B_8_^2−^ and B_9_^−^ molecular wheels, Wang and coworkers have suggested a general electronic design principle (*n* + *x* + *q* = *K*) for transition-metal centered boron molecular wheels M©B_*n*_^*q*−^,^26^ where *n* is the number of delocalized electrons supplied by the peripheral B_*n*_ ring, *x* is the formal valence of central metal atoms, *q* is the cluster's charge, and *K* is the total number of delocalized electrons. This electronic design principle is proved to be feasible in M©B_8_^−^ (M = Co, Re and Fe) and M©B_9_^−^ (M = Ru, Rh, Ir, Re and Fe) with *K* = 12.^17–19^ The deca-coordinated Ta©B_10_^−^ and Nb©B_10_^−^ molecular wheels have a total delocalized electrons of *K* = 16, possessing 10σ and 6π double aromaticity.^21^

In the transition-metal centred boron molecular wheels, the partly filled d-orbitals of transition-metal atoms play a crucial role in describing the interaction between the central metal atom and peripheral ring. In contrast, the metal atoms without d-orbital electrons (or with full-filled d-orbital electrons) don't like to form stable molecular wheel geometries. For instance, the AlB_7_^−^ and AlB_8_^−^ clusters are inclined to form the umbrella-shaped geometries.^27^ The Au atom with full-filled 5d-orbital electrons in AuB_10_^−^ cluster like forming a covalent B–Au σ bond with the corner B atom, serving as H atom. The 5d-orbital electrons, existing in the five lone pairs, could not effectively participate in bonding with the peripheral boron ring. The Au©B_10_^−^ molecular wheel is an extremely unstable local minimum (LM), being 1.95 eV higher in energy than the global minimum (GM) at B3LYP level.^28^ Beyond that, the cavity of peripheral ring need match up with the volume of central metal atom in physics. The small cavity cannot accommodate a metal atom. If the cavity is too large, the B–M interaction would be weakened dramatically due to the increased B–M distances. The molecular wheels will fail in competing with the half-sandwich structures.^29^ Therefore, it is a challenge to push the limit of coordination number in planar structures.

The present paper aims at breaking the record of deca-coordinated number in *D*_10h_ Ta©B_10_^−^ and Nb©B_10_^−^ molecular wheels, which is realized in the La©B_8_C_4_^+/0/−^ clusters. The title clusters have the interesting molecular wheel shapes, showing a central La atom encircled with a closed –(BCB)_4_– ring. The La©B_8_C_4_^+^ and La©B_8_C_4_ species have the *C*_4v_ symmetry with an out-of-plane distortion of La atoms, whereas the La©B_8_C_4_^−^ is perfectly plane. Thus, sequential reduction reaction in the La©B_8_C_4_^*q*^ (*q* = +1, 0, −1) results in the structural planarization. The chemical bonding analyses suggest that La©B_8_C_4_ and La©B_8_C_4_^−^ molecular wheels have 10σ and 9π/10π delocalized electrons, respectively, faithfully fulfilling the general double aromaticity rule. Whereas, the La©B_8_C_4_^+^ cluster is a counterexample in planar molecular wheels. It possesses 10σ and 8π delocalized electrons, being the first hypercoordinate molecular wheel with conflicting aromaticity.

## 2. Theoretical methods

We searched the GM structures for cationic and anionic La©B_8_C_4_^+/−^ clusters using the Coalescence Kick (CK) algorithm^30,31^ at B3LYP/LanL2DZ level, as well as the manual structural constructions. More than 3000 stationary points for each species were probed on their potential energy surfaces. The low-lying isomers (Δ*E* < 60 kcal mol^−1^) of La©B_8_C_4_^+/−^ and their corresponding neutral structures were reoptimized using B3LYP functional in Gaussian 09 package.^32^ The Stuttgart ECP28MWB_ANO basis set with the corresponding energy-consistent relativistic pseudopotential ECP28MWB was used for La, and 6-311+G* basis set for boron and carbon.^33^ This basis set combination is reasonable for current system according to the literature on transition-metal doped boron clusters.^34^ The vibrational frequencies were calculated at the same level to verify that the isomers presented are true minima. The relative energies of the top five lowest-lying isomers of La©B_8_C_4_^+/0/−^ were further refined at the single-point CCSD(T) level using the same basis set combination of B3LYP level.^35^ The top isomers of La©B_8_C_4_^*q*^ (*q* = +1, 0, −1) clusters are independently checked at the PBE0 level as well.

All electronic property calculations were performed at the same theory level with structural optimizations. The Wiberg bond indices (WBIs) and natural atomic charges of La©B_8_C_4_^*q*^ (*q* = +1, 0, −1) clusters were calculated using the NBO 6.0 program.^36^ The chemical bonding was elucidated using the canonical molecular orbital (CMO) analyses, the electron localization functions (ELFs)^37,38^ and adaptive natural density partitioning (AdNDP).^39^ Since the neutral La©B_8_C_4_ is an open-shell system, the AdNDP analysis is performed using the unrestricted AdNDP (UAdNDP) version. The anisotropy of current-induced density (ACID) analyses was carried out using ACID code.^40^ The ring-current images were visualized using the POV-Ray 3.7.^41^ and the ELFs and AdNDP data were visualized using Molekel 5.4.0.8.^42^

## 3. Results and discussion

### 3.1. The geometries and energies of La©B_8_C_4_^*q*^ (*q* = +1, 0, −1)

**Fig. 1:**
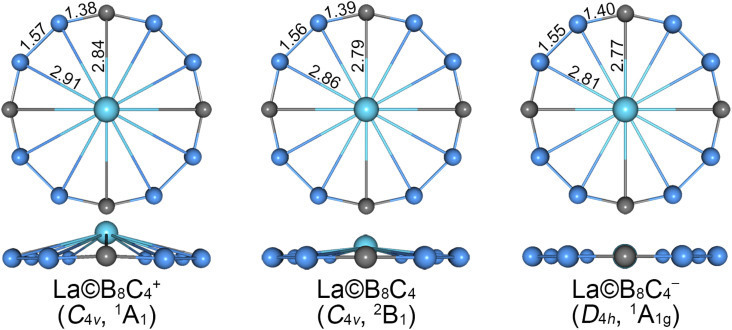
Optimized geometry for *C*_4v_ (^1^A_1_) global-minimum (GM) of La©B_8_C_4_^+^, *C*_4v_ (^2^B_1_) GM) of La©B_8_C_4_ and *D*_4h_ (^1^A_1g_) local-minimum (LM) of La©B_8_C_4_^−^ clusters at the B3LYP level. The bond distances are shown in Å.

The alternative low-lying isomers (sixty-seven structures) of cationic La©B_8_C_4_^+^ cluster are shown in Fig. S1 (ESI†). The GM structure, as shown in Fig. 1 and S1,† adopts an interesting molecular wheel style with a symmetry of *C*_4v_ (^1^A_1_), showing a dodeca-coordinated La atom surrounded by a fascinating –(BCB)_4_– ring. The central La atom has a little bulge with respect to the peripheral B_8_C_4_ ring. Its Cartesian coordinates are given in Table S1 (ESI†). The closest competitor has a symmetry of *C*_s_ (^1^A′), being 0.23 and 0.34 eV higher in energy than GM at single-point CCSD(T) and B3LYP levels, respectively, whose centered La atom is nona-coordinated with the B_5_C_4_ ring. The third and fourth isomers also adopt the molecular wheel structures, albeit with an inferior arrangement of B_8_C_4_ ring, which are at least 0.50 eV higher than GM at both levels. In particularly, the fifth isomer (*C*_4v_, ^3^A_1_) with a triplet ground state, possessing the same geometry with the GM, is 0.89 and 0.70 eV higher than GM at the CCSD(T) and B3LYP levels, respectively. Thus, the GM is clearly defined on its potential energy surface at the B3LYP and CCSD(T) levels.

The GM and low-lying isomers of neutral La©B_8_C_4_ cluster are presented in Fig. 1 and S2 (ESI†). All of them possess fascinating molecular wheel geometries. The GM (*C*_4v_, ^2^B_1_) of neutral La©B_8_C_4_ cluster adopts the similar architecture with cationic species, whose nearest competitor has a *C*_s_ (^2^A′) symmetry, being 0.19 and 0.18 eV higher in energy at the CCSD(T) and B3LYP levels.

As for anionic La©B_8_C_4_^−^ cluster, the potential energy surface appears to be more complicated. There are four isomeric geometries within 0.10 eV at the CCSD(T) and B3LYP levels (Fig. S3, ESI†), and the calculated energies are highly consistent at both levels. The GM structure of La©B_8_C_4_^−^ cluster adopts a *C*_s_ symmetry with the ^1^A′ electronic state, which is identical with that of the closest competitor of neutral La©B_8_C_4_ and the fourth isomer of cationic La©B_8_C_4_^+^. The perfectly planar *D*_4h_ (^1^A_1g_) isomer (Fig. 1) of La©B_8_C_4_^−^ cluster turns out to be a LM, which is only 0.04 eV higher in energy than GM at both CCSD(T) and B3LYP levels. We mainly focus on the perfect LM structure in this paper. The T_1_ diagnostic factors of CCSD(T) for three perfect molecular wheels are 0.022, 0.026 and 0.018, respectively, indicating the reliable CCSD(T) data.

### 3.2. The bond distances, Wiberg bond indices and natural atomic charges

The bond distances for GM (*C*_4v_, ^1^A_1_) of La©B_8_C_4_^+^ cluster are shown in Fig. 1. The GM has the equivalent B–B (1.57 Å) and B–C (1.38 Å) bond distances. The B–B bonds are distinctly shorter than the standard B–B single bond (1.70 Å),^43^ and being comparable with the B=B double bond (1.56 Å). The B–C bonds are even shorter than the B=C double bond (1.45 Å), being close to the B≡C triple bond (1.33 Å). The B–La and C–La bond distances are 2.91 and 2.84 Å, respectively. The central La atom is 0.70 Å out of B_8_C_4_ ring (not shown).

The GM (*C*_4v_, ^2^B_1_) of neutral La©B_8_C_4_ and LM (*D*_4h_, ^1^A_1g_) of La©B_8_C_4_^−^ are almost identical with the GM of La©B_8_C_4_^+^ species. The B–B and B–C bond distances of La©B_8_C_4_ are 1.56 and 1.39 Å, and those of La©B_8_C_4_^−^ are 1.55 and 1.40 Å, respectively, showing slight decrease for B–B bonds and increase for B–C bonds (within 0.01–0.02 Å) compared to La©B_8_C_4_^+^. Overall, the peripheral B_8_C_4_ ring enlarges by 0.04 Å for La©B_8_C_4_ and 0.08 Å for La©B_8_C_4_^−^ clusters compared with that of La©B_8_C_4_^+^ cluster. By contrast, the B–La and C–La bond distances shrink markedly (0.05–0.10 Å for the B–La bonds and 0.05–0.07 Å for C–La bonds). The B–La and C–La bond distances in La©B_8_C_4_ are 2.86 and 2.79 Å, and those in La©B_8_C_4_^−^ are 2.81 and 2.77 Å. The expansion of B_8_C_4_ ring and reduction of B–La/C–La links planarize the La©B_8_C_4_ and La©B_8_C_4_^−^ clusters. The out-of-plane distortion of central La reduces to 0.46 Å for La©B_8_C_4_. The La©B_8_C_4_^−^ turns into a perfectly planar structure.

The above bond distances are rationalized by the calculated WBIs (Fig. S4, ESI†) and natural charge (Fig. S5, ESI†) data from NBO analyses at the B3LYP level. In GM of La©B_8_C_4_^+^ cluster, the B–B and B–C bonds have the WBIs of 1.19 and 1.62, being distinctly greater than 1.0 (the latter in particular), implying that B_8_C_4_ ring is controlled by the delocalized σ/π bonds beyond the Lewis B–B/B–C two-center two-electrons (2c–2e) σ single bond. The covalent interaction of B–C bonds are markedly stronger than that of B–B ones, hinting that the delocalized electrons are mainly located at the B–C bonds of peripheral ring. Whereas, the covalent interaction between the central La and peripheral B_8_C_4_ ring is somewhat weak, with the small WBIs of 0.18 for B–La and 0.23 for C–La links. The WBIs of B–C, B–La and C–La bonds in neutral La©B_8_C_4_ and anion La©B_8_C_4_^−^ are consistent with those in La©B_8_C_4_^+^ cluster (within 0.01). The variations of WBIs among three structures are mainly occurred in B–B bonds, showing a gradual increase by 0.08. It implies that the extra one electron in La©B_8_C_4_ and two electrons in La©B_8_C_4_^−^ clusters are primarily dispersed on B–B bonds of B_8_C_4_ rings (see Section 3.4). Moreover, the total WBIs of centered La atom in La©B_8_C_4_^+/0/−^ clusters are 2.34, 2.41 and 2.45, respectively, indicating a negligible increase.

As for the natural charges (Fig. S5, ESI†), in La©B_8_C_4_^+^ cluster, the C atom carries a plentiful negative charge of −0.92 |*e*|, whereas the B atom is positively charged by +0.38 |*e*|, meaning the natural charges are markedly localized at C sites. Overall, the B_8_C_4_ ring has a collective negative charge of −0.64 |*e*|. It should be noted that the charge distribution on B_8_C_4_ ring is in line with their distinct electronegativity of B (2.04) and C (2.55) elements. The central La atom possesses a positive charge of +1.67 |*e*| with the electronic configuration of [Xe]6s^0.05^4f^0.10^5d^1.16^6p^0.01^5f^0.01^6d^0.02^, indicating an obvious charge transfer from the La atom to B_8_C_4_ ring, hinting a robust electrostatic attraction among them. The interaction between the central La and B_8_C_4_ ring is governed by both electrostatics and covalent interaction. In neutral La©B_8_C_4_ and anion La©B_8_C_4_^−^ clusters, the La atoms almost maintain the same positive charge with that in La©B_8_C_4_^+^. In contrast, the total charges of B_8_C_4_ rings increase to −1.64 |*e*| of La©B_8_C_4_ and −2.64 |*e*| of La©B_8_C_4_^−^. Specifically, the carrying charge of C atoms slightly decreases to −0.89 |*e*| of La©B_8_C_4_ and −0.86 |*e*| of La©B_8_C_4_^−^, and that of B atoms distinctly increases to +0.24 |*e*| and +0.10 |*e*|, which are corresponding with the bond distances and WBIs data of B–B/B–C bonds.

### 3.3. The stabilities of La©B_8_C_4_^+/0/−^ along with out-of-plane distortion of La atom

**Fig. 2:**
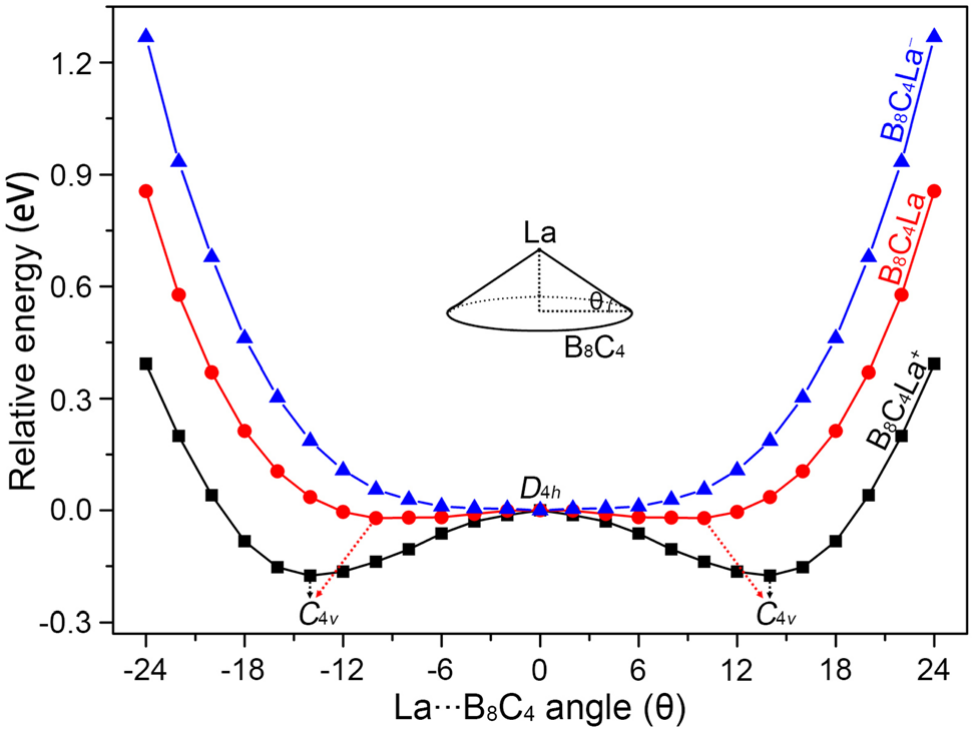
The potential energy curves of *C*_4v_ (^1^A_1_) GM La©B_8_C_4_, *C*_4v_ (^2^B_1_) GM La©B_8_C_4_, and *D*_4h_ (^1^A_1g_) LM La©B_8_C_4_^−^ clusters along with the out-of-plane distortion central La atom.

The B_8_C_4_ rings of La©B_8_C_4_^+^ and La©B_8_C_4_ are slightly small, cannot accommodate the large La atom, leading to a tiny hump of central La atom. In order to quantitatively evaluate the relative stabilities of La©B_8_C_4_^+/0/−^ molecular wheels with different La height relative to the B_8_C_4_ rings, we performed the potential energy scanning as the La atoms move along with their *C*_4_ axes, prescribed by the La⋯B_8_C_4_ angle *θ*, as shown in Fig. 2. The perfect *D*_4h_ structure of La©B_8_C_4_^+^ is a one-order saddle point with the imaginary frequency of 73.93i cm^−1^, which is 0.17 eV above the *C*_4v_ GM La©B_8_C_4_^+^. In essence, the *D*_4h_ structure is the transition state (TS) structure for the La atom traversing the B_8_C_4_ ring. The *C*_4v_ GM of La©B_8_C_4_^+^ has the *θ* value of ±14.19°, being corresponding to a La height of 0.70 Å above the center of B_8_C_4_ ring. The potential energy curve of La©B_8_C_4_ is flatter than that of La©B_8_C_4_^+^, whose *D*_4h_ structure (with a small imaginary frequency of 49.82i cm^−1^) is only 0.03 eV higher in energy than *C*_4v_ GM of La©B_8_C_4_, which is attribute to its enlarged B_8_C_4_ ring and reduced B–La/C–La bond distances. The potential energy curve of La©B_8_C_4_^−^ indicates that the *D*_4h_ structure is the true minimum.

### 3.4. Chemical bonding

In view of the similar molecular wheel structures of La©B_8_C_4_^+/0/−^ clusters, the chemical bonding analyses are mainly focused on the close-shelled La©B_8_C_4_^+^ and La©B_8_C_4_^−^ species. The La©B_8_C_4_^+^ cluster has 42 valence electrons, occupying 21 valence CMOs (Fig. S6, ESI†). These occupied CMOs are divided into three subsets based on their constituent atomic orbitals (AOs). The twelve CMOs in subset (a) are primarily composed of 2s and tangential 2p AOs of B/C, which can be reasonably localized into eight 2c–2e B–C and four 2c–2e B–B Lewis σ bonds in B_8_C_4_ ring. The five delocalized σ CMOs in subset (b) are composed of the radial 2p AOs of B/C and 5d AOs of La (d_*x*^2^−*y*^2^_ in HOMO and d_*xy*_ in HOMO-2). It should be noted that the HOMO and HOMO-2 σ CMOs are formally degenerated. These five CMOs constitute the delocalized σ framework, resulting in the 10σ aromaticity of La©B_8_C_4_^+^, according to (4*N*_σ_ + 2) Hückel rule (*N*_σ_ = 2). There are four delocalized π CMOs in subset (c), being composed of the vertical 2p AOs of B/C and 5d AOs of La (d_*xz*_ in HOMO-4 and d_*yz*_ in HOMO-4′), constructing the π framework of La©B_8_C_4_^+^, and leading to its 8π antiaromaticity in view of (4*N*_π_) Hückel rule (*N*_π_ = 2). Overall, La©B_8_C_4_^+^ molecular wheel is a system of 10σ and 8π conflicting aromaticity. Following Boldyrev, the term conflicting aromaticity refers to the systems with simultaneous presence of aromaticity and antiaromaticity in orthogonal planes, there is aromaticity in one plane and anti-aromaticity in the plane orthogonal to it.^14,44^ In nature, the four delocalized π CMOs can be appropriately recombined four three-center two-electrons (3c–2e) –BCB– island π bonds (see the ELFs and AdNDP analyses), being in line with its *C*_4v_ symmetry.

The La©B_8_C_4_^−^ cluster is a system with 44 valence electrons, occupying 22 CMOs (Fig. S7, ESI†). It has one more full-filled π CMO (HOMO) than La©B_8_C_4_^+^. The five delocalized π CMOs in subset (c) are one-to-one corresponding with the σ CMOs in subset (b), following the same spatial distribution. Thus, La©B_8_C_4_^−^ cluster is double aromatic with 10σ and 10π delocalized electrons. The σ CMOs (HOMO-1/HOMO-4) and π CMOs (HOMO/HOMO-2) also are formally degenerated, being similar to the σ CMOs (HOMO/HOMO-2) of La©B_8_C_4_^+^ in Fig. S6(b) (ESI†). The energy gap between the HOMO and HOMO-2 is 1.83 eV, which hints the out-of-plane distortion of central La atom in neutral La©B_8_C_4_ and cationic La©B_8_C_4_^+^ molecular wheels is not due to the Jahn–Teller effect. The electron density of HOMO is mainly dispersed on four B–B bonds, which leads to decrease of bond distances by 0.02 Å (Fig. 1) and increase of WBIs by 0.16 compared with those in La©B_8_C_4_^+^ wheel (Fig. S4, ESI†).

**Fig. 3:**
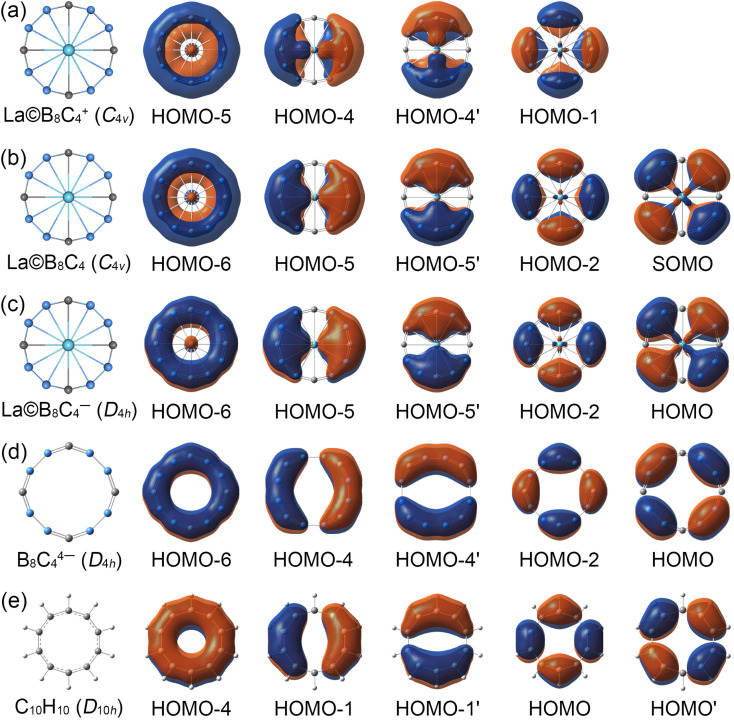
Comparison of the delocalized canonical molecular orbitals (CMOs) between (a) *C*_4v_ (^1^A_1_) GM La©B_8_C_4_, (b) *C*_4v_ (^2^B_1_) GM La©B_8_C_4_, (c) *D*_4h_ (^1^A_1g_) LM La©B_8_C_4_^−^ clusters, (d) *D*_4h_ (^1^A_1g_) B_8_C_4_^4−^ and (e) *D*_10h_ C_10_H_10_ (annulene) model molecule.

The CMOs bonding pattern of La©B_8_C_4_ cluster (Fig. S8, ESI†) is similar to that of La©B_8_C_4_^−^ cluster, albeit possessing a single occupied π molecular orbital (that is the SOMO). Thus, La©B_8_C_4_ molecular wheel is dominated by 10σ and 9π double aromaticity. The delocalized π frameworks in La©B_8_C_4_^+/0/−^ clusters are compared with the *D*_4h_ B_8_C_4_^4−^ ring and planar aromatic *D*_10h_ C_10_H_10_ (annulene) model molecule (see Fig. 3). Note that the *D*_4h_ B_8_C_4_^4−^ ring and *D*_10h_ C_10_H_10_ are not the true minimums.^45^ The delocalized π CMOs in La©B_8_C_4_ and La©B_8_C_4_^−^ clusters are one to one corresponding to those in *D*_4h_ B_8_C_4_^4−^ ring and *D*_10h_ C_10_H_10_ model. Importantly, the SOMO in La©B_8_C_4_ and HOMO in La©B_8_C_4_^−^ facilitate the π electron delocalization on B_8_C_4_ ring and enhance the interaction of B–La links, which would obviously shorten the B–La bond distances, and make the molecular wheels planarization.

It should be pointed out that the coordination interaction between the La atom and B_8_C_4_ ring is mainly attributed to the d–p (La 5d_*x*^2^−*y*^2^_/5d_*xy*_ AOs and B/C radial 2p AOs) σ CMOs (HOMO/HOMO-2 in La©B_8_C_4_^+^ and HOMO-1/HOMO-4 in La©B_8_C_4_^−^) and d–p (La 5d_*xz*_/5d_*yz*_ AOs and B/C vertical 2p AOs) π CMOs (HOMO-4/4′ in La©B_8_C_4_^+^ and HOMO-5/5′ in La©B_8_C_4_^−^). The coordination interactions of d–p σ CMOs with the relatively large orbital component of La 5d AOs are a little bit stronger than those of d–p π CMOs. Quantitively, the HOMO and HOMO-2 in La©B_8_C_4_^+^ (Fig. S6, ESI†) have a 19.89% La 5d_*x*^2^−*y*^2^_ and 10.39% La 5d_*xy*_ AOs contribution, respectively. In La©B_8_C_4_^−^, the La 5d_*x*^2^−*y*^2^_ AO contributes by 14.58% to HOMO-1, and La 5d_*xy*_ AO contributes by 8.42% to HOMO-4 (Fig. S7, ESI†). For the d–p π CMOs, the La 5d_*xz*_/5d_*yz*_ AOs contributes merely by 6.72% in HOMO-4/4′ of La©B_8_C_4_^+^, and 8.11% in HOMO-5/5′ of La©B_8_C_4_^−^.

**Fig. 4:**
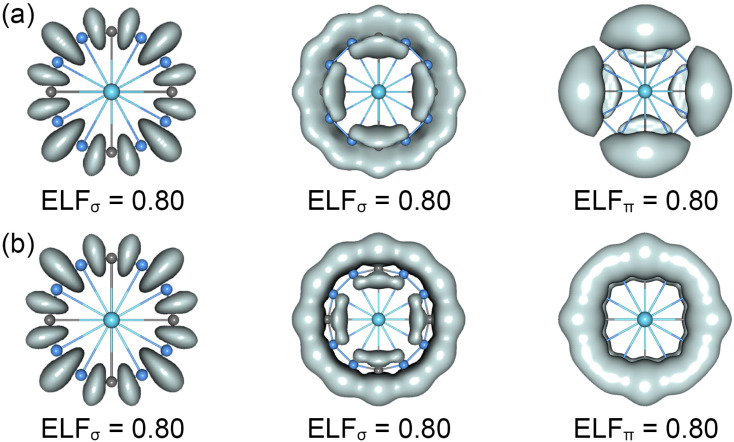
Electron localization functions (ELFs) for (a) the *C*_4v_ (^1^A_1_) GM of La©B_8_C_4_^+^ and (b) the *D*_4h_ (^1^A_1g_) LM of La©B_8_C_4_^−^ clusters.

The CMOs bonding images of La©B_8_C_4_^+/−^ molecular wheels are faithfully confirmed by the ELFs analyses. As shown in Fig. 4, the ELF_σ_ patterns of La©B_8_C_4_^+^ and La©B_8_C_4_^−^ clusters are almost identical, showing twelve localized 2c–2e B–B/B–C σ bonds (left panels) and strong global delocalized σ electron density (middle panels). Obviously, two molecular wheels mainly differ in ELF_π_ patterns. The π electron density of La©B_8_C_4_^+^ is clearly segmented into four –BCB– islands (right panels), but that of La©B_8_C_4_^−^ is continuous, and being smoothly distributed on the whole B_8_C_4_ ring, which firmly supports the above assessments of 8π antiaromaticity for La©B_8_C_4_^+^ and 10π aromaticity for La©B_8_C_4_^−^.

**Fig. 5:**
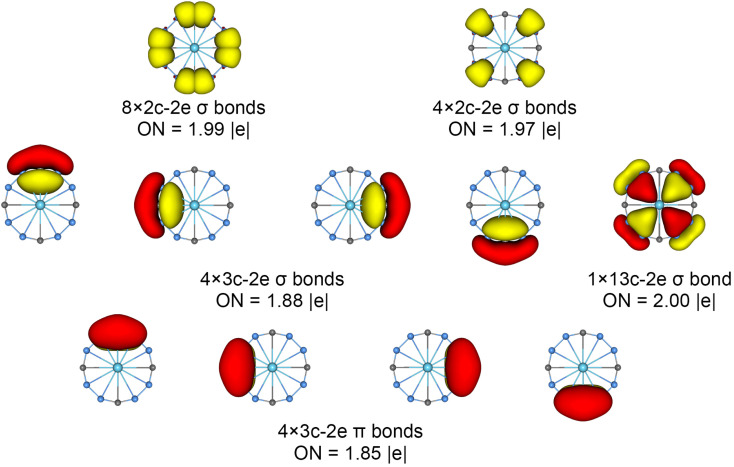
Chemical bonding pattern of the *C*_4v_ (^1^A_1_) GM La©B_8_C_4_^+^ cluster based on the adaptive natural density partitioning (AdNDP) analysis. Occupation numbers (ONs) are indicated.

**Fig. 6:**
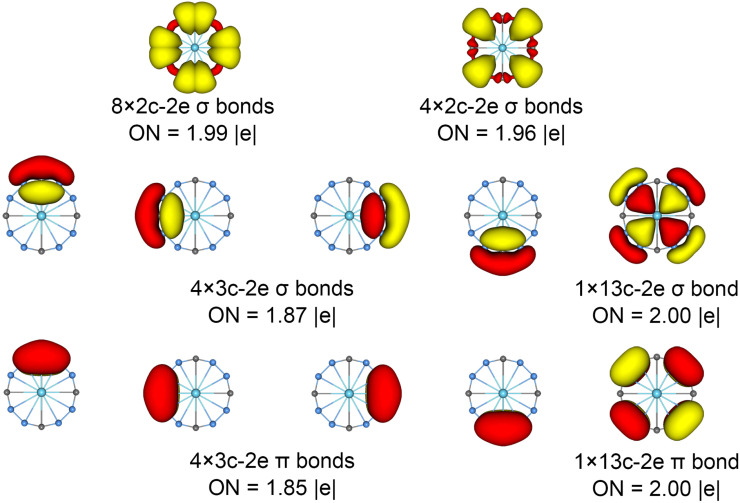
Chemical bonding pattern of the *D*_4h_ (^1^A_1g_) LM La©B_8_C_4_^−^ cluster based on the AdNDP analysis. The ONs are indicated.

The chemical bonding patterns in both La©B_8_C_4_^+/−^ molecular wheels are accurately elucidated by the AdNDP analyses. The AdNDP results for La©B_8_C_4_^+^ (Fig. 5) reveal eight localized 2c–2e B–C σ bonds and four localized 2c–2e B–B σ bonds in peripheral B_8_C_4_ ring, which are related to the CMOs in Fig. S6(a) (ESI†). There are four 3c–2e –BCB– island σ bonds and one thirteen-center two-electrons (13c–2e) σ bond in B_8_C_4_ ring, supporting its 10σ aromaticity. Meanwhile, the four 3c–2e –BCB– island π bonds are in line with 8π antiaromaticity. The island version for four 3c–2e σ and π bonds are fully supported by the ELFs analyses. The AdNDP results for La©B_8_C_4_^−^ (Fig. 6) show the identically localized B–B/B–C σ bonds and delocalized σ framework on B_8_C_4_ ring with La©B_8_C_4_^+^. Differently, it has one more fully delocalized 13c–2e π bond except for four 3c–2e –BCB– island π bonds, firmly confirmed its 10π aromaticity. It should be noted that the occupation numbers (ON) of the 3c–2e –BCB– σ and π bonds in both La©B_8_C_4_^+^ and La©B_8_C_4_^−^ clusters (1.88/1.87 *versus* 1.85) are less than 2, hinting a small degree of covalent interaction between the B_8_C_4_ ring and the central La atom, which is consistent with the small WBIs of B–La/C–La links. The AdNDP data of La©B_8_C_4_ cluster (obtained from UAdNDP version) are indicated in Fig. S9 (ESI†), showing one global delocalized 13c–1e π bond. In addition, the AdNDP chemical bonding images (four 3c–2e island σ/π bonds plus one 13c–2e delocalized σ/π bond) of La©B_8_C_4_^+/−^ molecular wheels are reasonable, which is in line with the structural data, and rationalizes the delocalized σ and π frameworks as well.

The anisotropy of current-induced density (ACID) analyses was performed to probe the ring currents of La©B_8_C_4_^+^ and La©B_8_C_4_^−^ clusters, induced by an external magnetic field being vertical to molecular wheels. The decomposed σ and π-ring current images with a high-resolution are depicted in Fig. S10 (ESI†). The La©B_8_C_4_^+^ molecular wheel shows the robust σ-ring current (Fig. S10(a)†) on whole B_8_C_4_ peripheral ring. In contrast, its π-ring current is clearly localized on four –BCB– units, showing local circulation for vector of induced current density. The direction of π-ring current is opposite to that of σ-ring currents, supporting the 10σ and 8π conflicting aromaticity. The La©B_8_C_4_^−^ molecular wheel shows the robust σ and π-ring current (Fig. S10(b)†), perfectly elucidating the 10σ and 10π double aromaticity.

The thermal stabilization for La©B_8_C_4_^+/0/−^ molecular wheels is mainly attributed to the cyclic electron delocalization in peripheral B_8_C_4_ ring, albeit the La©B_8_C_4_^+^ cluster possesses 8π antiaromaticity. Furthermore, we calculated the bond dissociation energy (BDE) and inherent interaction energy of La©B_8_C_4_ between the central La atom and B_8_C_4_ motif according to the below formulas.La©B_8_C_4_ (*C*_4v_, ^2^B_1_) = B_8_C_4_ (LM, *D*_4h_, ^1^A_1g_) + LaLa©B_8_C_4_ (*C*_4v_, ^2^B_1_) = B_8_C_4_ (frozen, *D*_4h_, ^1^A_1g_) + Lawhere, the B_8_C_4_ (LM, *D*_4h_, ^1^A_1g_) has a square geometry, a true local minimum on its potential surface. Rather, the B_8_C_4_ (frozen, *D*_4h_, ^1^A_1g_) represents the frozen fragment form La©B_8_C_4_ molecular wheel, which is a second-order saddle point. The B_8_C_4_ (LM, *D*_4h_, ^1^A_1g_) and B_8_C_4_ (frozen, *D*_4h_, ^1^A_1g_) are calculated at B3LYP/6-311+G* level, the La atom is done at B3LYP/ECP28MWB_ANO level, respectively. The BDE and inherent interaction energies are as high as −178.99 and −208.59 kcal mol^−1^, respectively, confirming the stabilization of dodeca-coordinated La©B_8_C_4_ molecular wheel. The energy difference between the BDE and inherent interaction energy describes the isomerization energy of B_8_C_4_ motif from the square geometry to a circular one in nature (Fig. S11, ESI†).

### 3.5. The electronic counting rule in La©B_8_C_4_^*q*^ molecular wheels

The fascinating transition-metal centered boron molecular wheels M©B_*n*_^*q*−^ is controlled by the double (σ and π) aromaticity, following the electronic design principle (*n* + *x* + *q* = *K*) proposed by Wang and coworkers.^26^ In intriguing La©B_8_C_4_^+/0/−^ boron–carbon molecular wheels, each C atom can provide two delocalized electrons, one more than B atom. Thus, an update version of electronic design principle for M©B_*m*_C_*n*_^*q*−^ molecular wheels is proposed, that is *m* + 2*n* + *x* ± *q* = *K*, where the *m* and *n* is the number of peripheral B and C atoms, *x* is the formal valence of central metal atoms, *q* is the cluster's charge. The La©B_8_C_4_^+/0/−^ molecular wheels are unprecedent with the highest dodeca-coordination number in plane, following *m* + 2*n* + *x* ± *q* = 18, 19 and 20 electronic counting principles, respectively. The anionic La©B_8_C_4_^−^ molecular wheel has (10σ + 10π) double aromaticity, following (4*N*_σ/π_ + 2) Hückel rule with *N*_σ_ = *N*_π_ = 2, respectively. The neutral La©B_8_C_4_ molecular wheel is an open-shell system with a single occupied π orbital, possessing the 10σ and 9π double aromaticity. Interestingly, the cationic La©B_8_C_4_^+^ cluster is a conflicting aromatic system of with 10σ and 8π delocalized electrons (*K* = 18), obeying the (4*N*_σ_ + 2) and (4*N*_π_) Hückel rule with *N*_σ_ = *N*_π_ = 2, respectively. Thus, La©B_8_C_4_^+^ represents a counterexample for double aromaticity in planar hypercoordinate molecular wheels.

## 4. Conclusions

In summary, we have theoretically predicated three dodeca-coordinated La©B_8_C_4_^+/0/−^ molecular wheels, featuring a central La atom enclosed by a perfectly planar B_8_C_4_ ring, which is viable in the –(BCB)_4_– form. The La©B_8_C_4_^+^ and La©B_8_C_4_ molecular wheels have the *C*_4v_ symmetry with a slightly out-of-plane distortion of La atom. The La©B_8_C_4_^−^ molecular wheel is perfectly planar. The stabilizations of La©B_8_C_4_^+/0/−^ molecular wheels are benefit from their unique chemical bonding. The La©B_8_C_4_ and La©B_8_C_4_^−^ molecular wheels have 10σ and 9π/10π delocalized electrons, obeying the universal principle of double aromaticity, whereas, the La©B_8_C_4_^+^ molecular wheel is a counterexample, which has the conflicting aromaticity with 10σ and 8π delocalized electrons. The La©B_8_C_4_^+/0/−^ molecular wheels with dodeca-coordination number are unprecedented for a planar system in coordination chemistry.

## Conflicts of interest

There are no conflicts to declare.

## Supplementary Material

RA-013-D2RA07155J-s001
