# Supplementary material for: The dodeca-coordinated La©B8C4+/0/− molecular wheels: conflicting aromaticity versus double aromaticity
Source: RSC Adv. 2023 Jan 19;13(5):3071–8. doi: 10.1039/d2ra07155j (PMC9850364; doi:10.1039/d2ra07155j)
Supplement: RA-013-D2RA07155J-s001 [file RA-013-D2RA07155J-s001.pdf]

Electronic Supplementary Material (ESI) for RSC Advances.
This journal is © The Royal Society of Chemistry 2023

SUPPLEMENTARY INFORMATION

The Dodeca-Coordinated La©$B_8C_4^{+/0/-}$ Molecular Wheels: Conflicting Aromaticity versus Double Aromaticity†

Ying-Jin Wang,* Jia-Xin Zhao, Miao Yan, Lin-Yan Feng, Chang-Qing Miao, and Cheng-Qi Liu

Department of Chemistry, Xinzhou Teachers University, Xinzhou 034000, Shanxi, China

*E-mail: yingjinwang@sxu.edu.cn

Supplementary Information

Table S1. Cartesian coordinates for the C_{4v} ($^1A_1$) GM La©$B_8C_4^+$, C_{4v} ($^2B_1$) GM La©B_8C_4, and D_{4h} ($^1A_{1g}$) LM La©$B_8C_4^-$ clusters at B3LYP level.

Figure S1. Alternative optimized Low-lying isomers of La©$B_8C_4^+$ cluster. The relative energies are shown at the single-point CCSD(T) (for top five), B3LYP (in square brackets) and PBE0 (in curly brackets) levels, respectively. The relative energies at the B3LYP and PBE0 levels are corrected with the zero-point energies (ZPEs). All energies are in eV.

Figure S2 The top eleven Low-lying isomers of La©B_8C_4 cluster. The relative energies are shown at the single-point CCSD(T) (for top five), B3LYP (in square brackets) and PBE0 (in curly brackets) levels, respectively. The relative energies at the B3LYP and PBE0 levels are corrected with the zero-point energies (ZPEs). All energies are in eV.

Figure S3 The top ten Low-lying isomers of La©$B_8C_4^-$ cluster. The relative energies are shown at the single-point CCSD(T) (for top five), B3LYP (in square brackets)

and PBE0 (in curly brackets) levels, respectively. The relative energies at the B3LYP and PBE0 levels are corrected with the zero-point energies (ZPEs). All energies are in eV.

Figure S4. Calculated Wiberg bond indices (WBIs) of the C_{4v} ($^1A_1$) GM La©$B_8C_4^+$, C_{4v} GM ($^2B_1$) La©B_8C_4 and D_{4h} ($^1A_{1g}$) LM La©$B_8C_4^-$ clusters from the natural bond orbital (NBO) analyses at B3LYP level.

Figure S5. Calculated natural atomic charges of the C_{4v} ($^1A_1$) GM La©$B_8C_4^+$, GM C_{4v} ($^2B_1$) La©B_8C_4 and D_{4h} LM ($^1A_{1g}$) La©$B_8C_4^-$ clusters from the NBO analyses at B3LYP level.

Figure S6. Occupied canonical molecular orbitals (CMOs) of the C_{4v} ($^1A_1$) GM La©$B_8C_4^+$ cluster. (a) Twelve σ CMOs for two-center two-electron (2c-2e) B−C/B−B Lewis σ bonds in B_8C_4 ring. (b) Five delocalized σ CMOs in La©$B_8C_4^+$. (c) Four delocalized π CMOs in La©$B_8C_4^+$.

Figure S7. Occupied canonical molecular orbitals (CMOs) of the D_{4h} ($^1A_{1g}$) LM La©$B_8C_4^-$ cluster. (a) Twelve σ CMOs for 2c-2e B−C/B−B Lewis σ bonds in B_8C_4 ring. (b) Five delocalized σ CMOs in La©$B_8C_4^-$. (c) Five delocalized π CMOs in La©$B_8C_4^-$.

Figure S8. Occupied canonical molecular orbitals (CMOs) of the C_{4v} ($^2B_1$) GM La©B_8C_4 cluster. (a) Twelve σ CMOs for 2c-2e B−C/B−B Lewis σ bonds in B_8C_4 ring. (b) Five delocalized σ CMOs in La©B_8C_4. (c) Five delocalized π CMOs in La©B_8C_4, the SOMO represents single occupation.

Figure S9. Chemical bonding pattern of the C_{4v} ($^2B_1$) GM La©B_8C_4 cluster based on the unrestricted adaptive natural density partitioning (UAdNDP) analysis. Occupation numbers (ONs) are indicated.

Figure S10. The calculated σ and π-ring current images of C_{4v} ($^1A_1$) GM La©$B_8C_4^+$ and D_{4h} ($^1A_{1g}$) LM La©$B_8C_4^-$ clusters. The external magnetic field is perpendicular to the molecular wheels.

Figure S11. The energy cycle of C_{4v} ($^2B_1$) GM La©B_8C_4 cluster, along with the isomerization energy of B_8C_4 ring, bond dissociation energy (BDE) and inherent interaction energy between the central Y and B_8C_4 ring (in kcal mol^{-1}).

Figure S1. Alternative optimized Low-lying isomers of La©$B_8C_4^+$ cluster. The relative energies are shown at the single-point CCSD(T) (for top five), B3LYP (in square brackets) and PBE0 (in curly brackets) levels, respectively. The relative energies at the B3LYP and PBE0 levels are corrected with the zero-point energies (ZPEs). All energies are in eV.

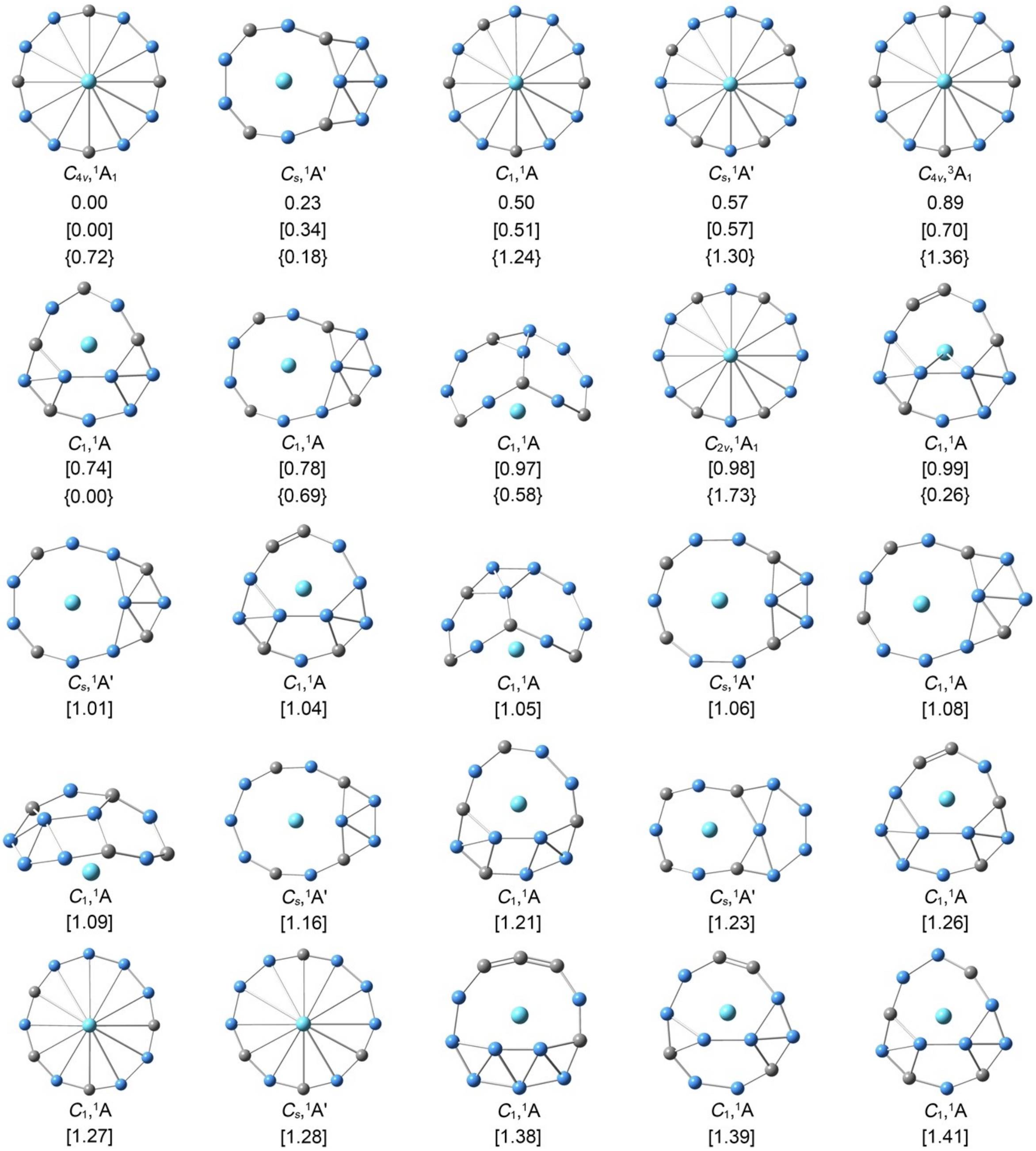

Continue ……

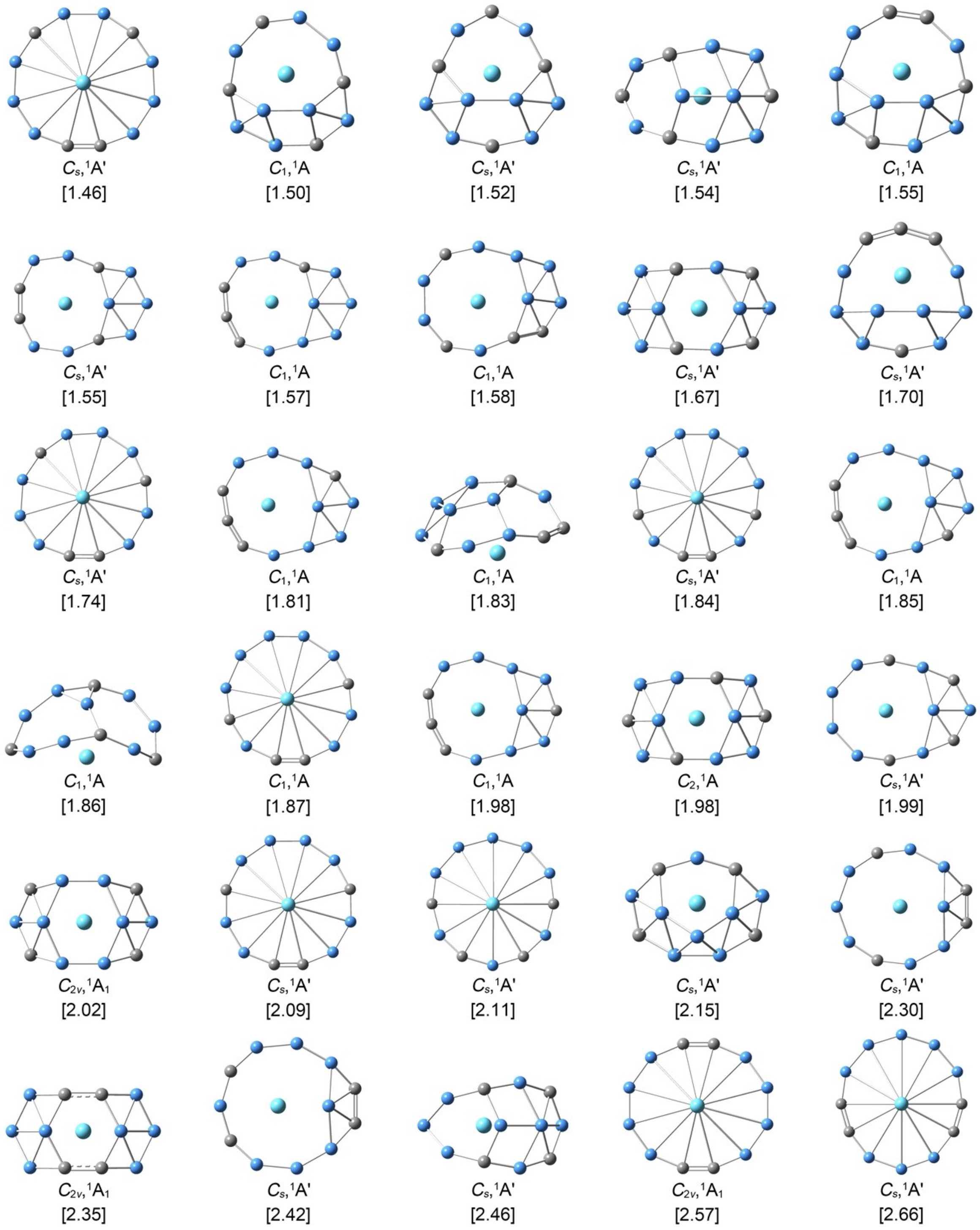

C_s,^{1}A' [1.46]
C_1,^{1}A [1.50]
C_s,^{1}A' [1.52]
C_s,^{1}A' [1.54]
C_1,^{1}A [1.55]
C_s,^{1}A' [1.55]
C_1,^{1}A [1.57]
C_1,^{1}A [1.58]
C_s,^{1}A' [1.67]
C_s,^{1}A' [1.70]
C_s,^{1}A' [1.74]
C_1,^{1}A [1.81]
C_1,^{1}A [1.83]
C_s,^{1}A' [1.84]
C_1,^{1}A [1.85]
C_1,^{1}A [1.86]
C_1,^{1}A [1.87]
C_1,^{1}A [1.98]
C_2,^{1}A [1.98]
C_s,^{1}A' [1.99]
C_{2v},$^1A_1$ [2.02]
C_s,^{1}A' [2.09]
C_s,^{1}A' [2.11]
C_s,^{1}A' [2.15]
C_s,^{1}A' [2.30]
C_{2v},$^1A_1$ [2.35]
C_s,^{1}A' [2.42]
C_s,^{1}A' [2.46]
C_{2v},$^1A_1$ [2.57]
C_s,^{1}A' [2.66]

Continue ……

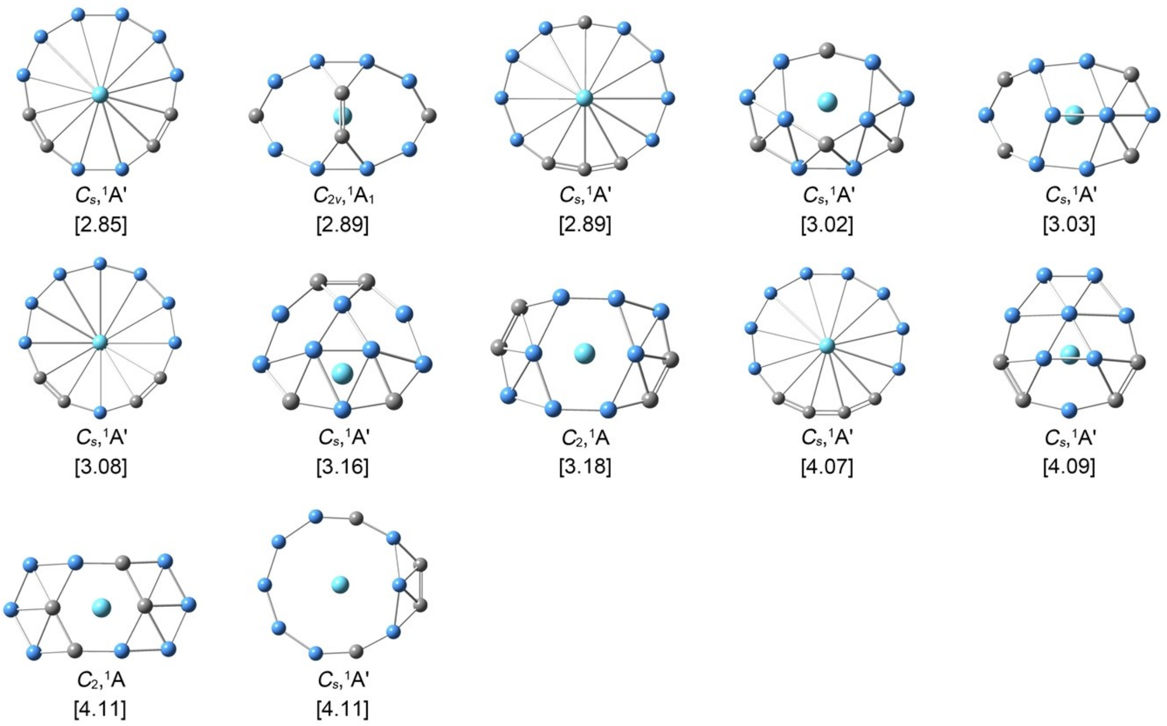

C_s, ^{1}A'
[2.85]
C_{2v}, ^{1}A$_1$
[2.89]
C_s, ^{1}A'
[2.89]
C_s, ^{1}A'
[3.02]
C_s, ^{1}A'
[3.03]
C_s, ^{1}A'
[3.08]
C_s, ^{1}A'
[3.16]
C_2, ^{1}A
[3.18]
C_s, ^{1}A'
[4.07]
C_s, ^{1}A'
[4.09]
C_2, ^{1}A
[4.11]
C_s, ^{1}A'
[4.11]

Figure S2 The top eleven Low-lying isomers of La©B_8C_4 cluster. The relative energies are shown at the single-point CCSD(T) (for top five), B3LYP (in square brackets) and PBE0 (in curly brackets) levels, respectively. The relative energies at the B3LYP and PBE0 levels are corrected with the zero-point energies (ZPEs). All energies are in eV.

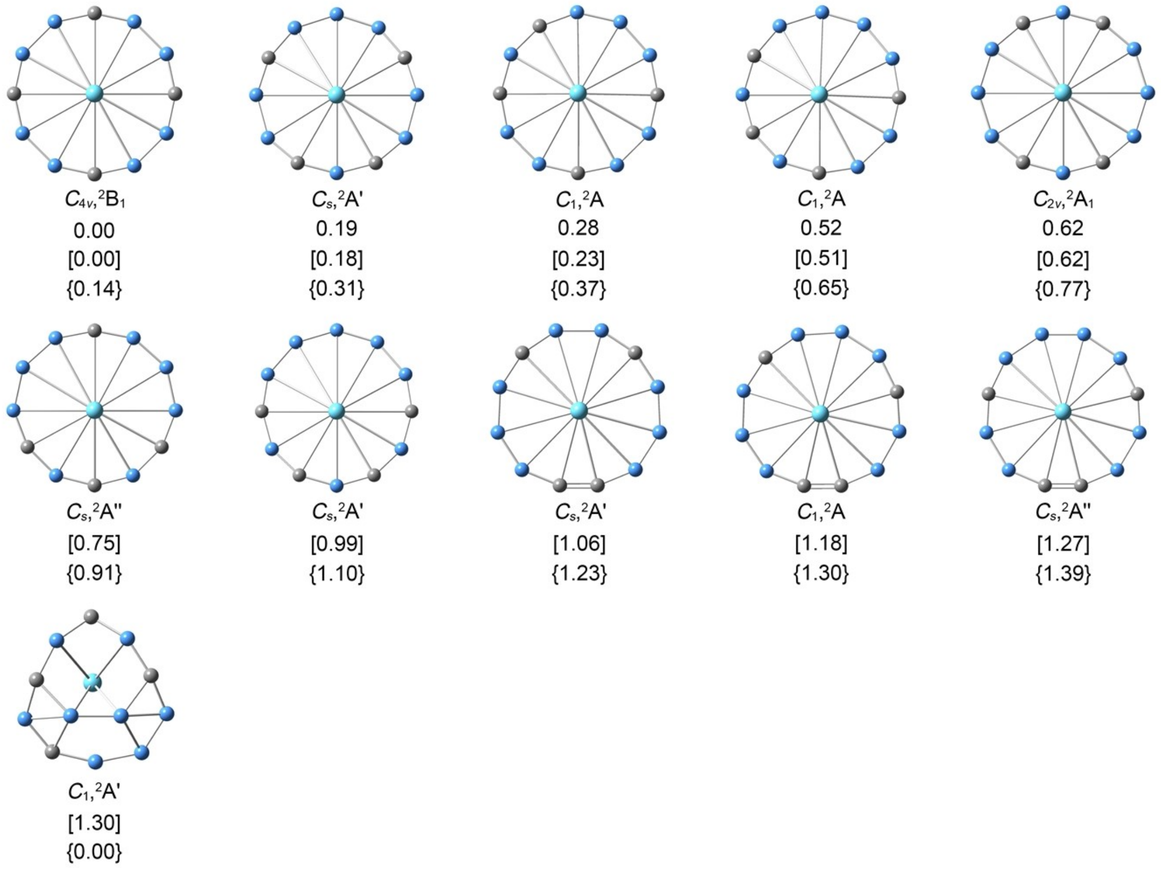

Figure S3 The top ten Low-lying isomers of La©$B_8C_4^-$ cluster. The relative energies are shown at the single-point CCSD(T) (for top five), B3LYP (in square brackets) and PBE0 (in curly brackets) levels, respectively. The relative energies at the B3LYP and PBE0 levels are corrected with the zero-point energies (ZPEs). All energies are in eV.

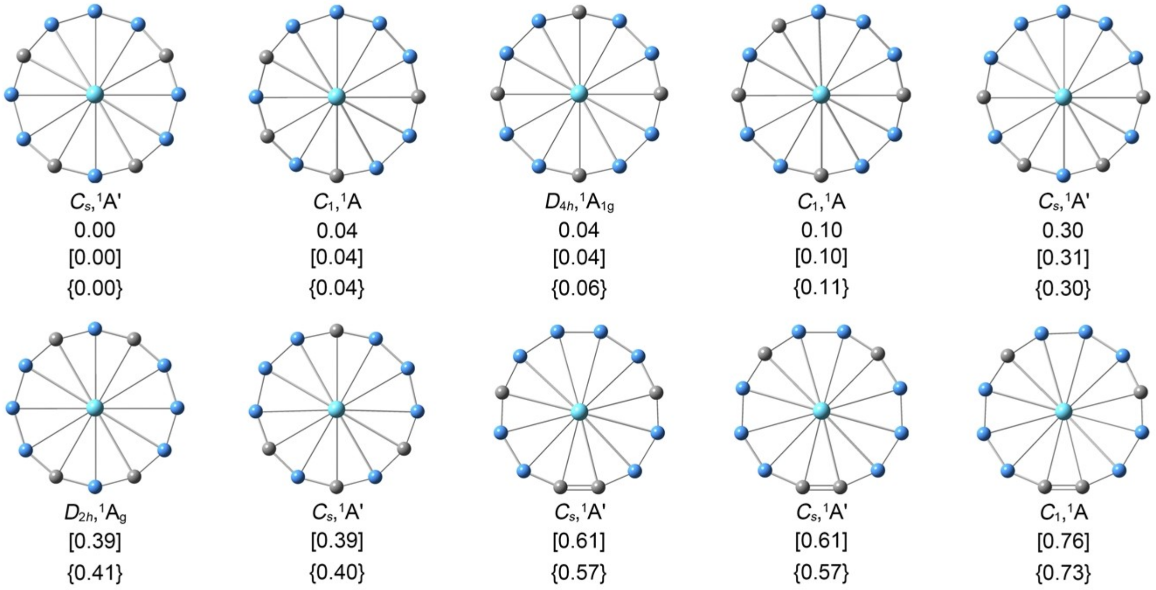

Figure S4. Calculated Wiberg bond indices (WBIs) of the C_{4v} ($^1A_1$) GM La©$B_8C_4^+$, C_{4v} GM ($^2B_1$) La©B_8C_4 and D_{4h} ($^1A_{1g}$) LM La©$B_8C_4^-$ clusters from the natural bond orbital (NBO) analyses at B3LYP level.

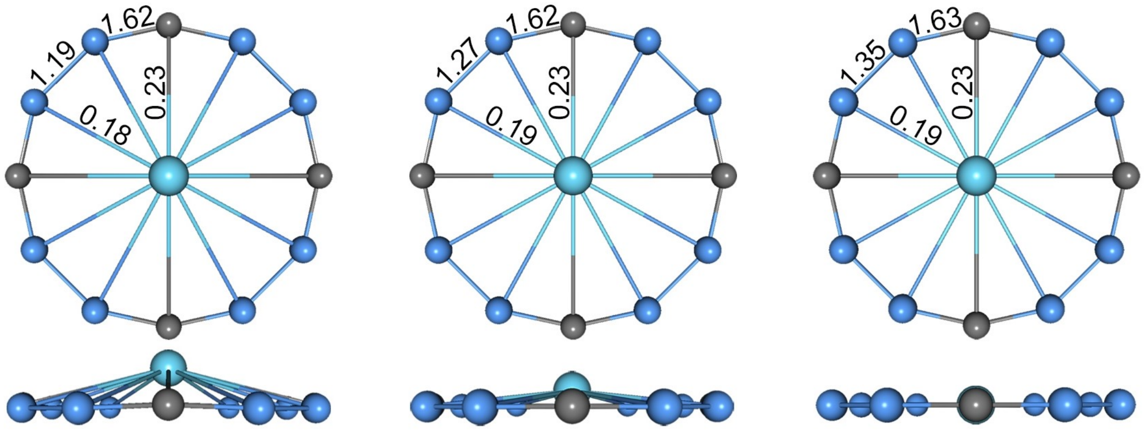

La©$B_8C_4^+$ (C_{4v}, $^1A_1$)

La©B_8C_4 (C_{4v}, $^2B_1$)

La©$B_8C_4^-$ (D_{4h}, $^1A_{1g}$)

Figure S5. Calculated natural atomic charges of the C_{4v} ($^1A_1$) GM La©$B_8C_4^+$, GM C_{4v} ($^2B_1$) La©B_8C_4 and D_{4h} LM ($^1A_{1g}$) La©$B_8C_4^-$ clusters from the natural bond orbital (NBO) analyses at B3LYP level.

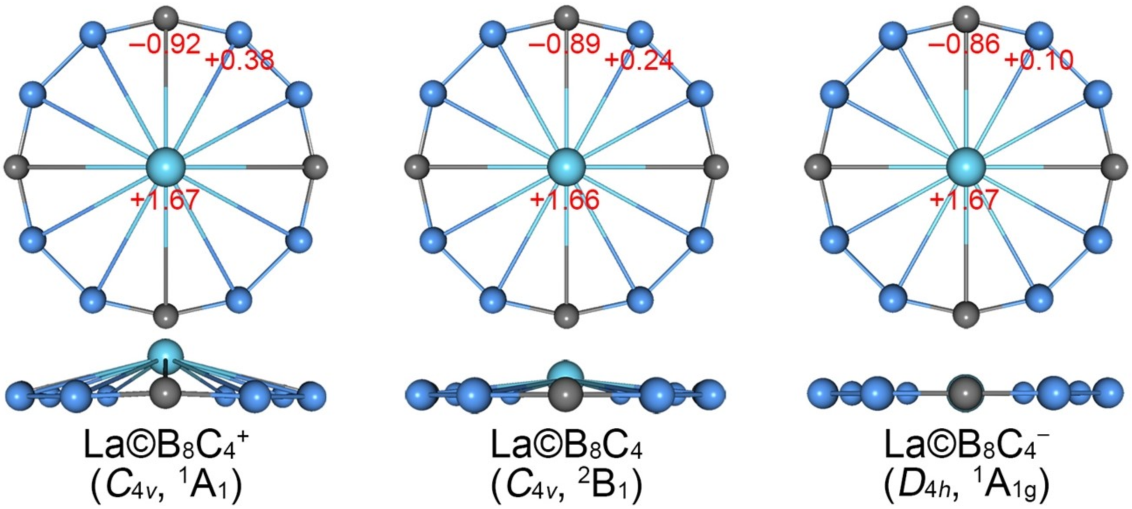

Figure S6. Occupied canonical molecular orbitals (CMOs) of the C_{4v} ($^1A_1$) GM La©$B_8C_4^+$ cluster. (a) Twelve σ CMOs for two-center two-electron (2c-2e) B−C/B−B Lewis σ bonds in B_8C_4 ring. (b) Five delocalized σ CMOs in La©$B_8C_4^+$. (c) Four delocalized π CMOs in La©$B_8C_4^+$.

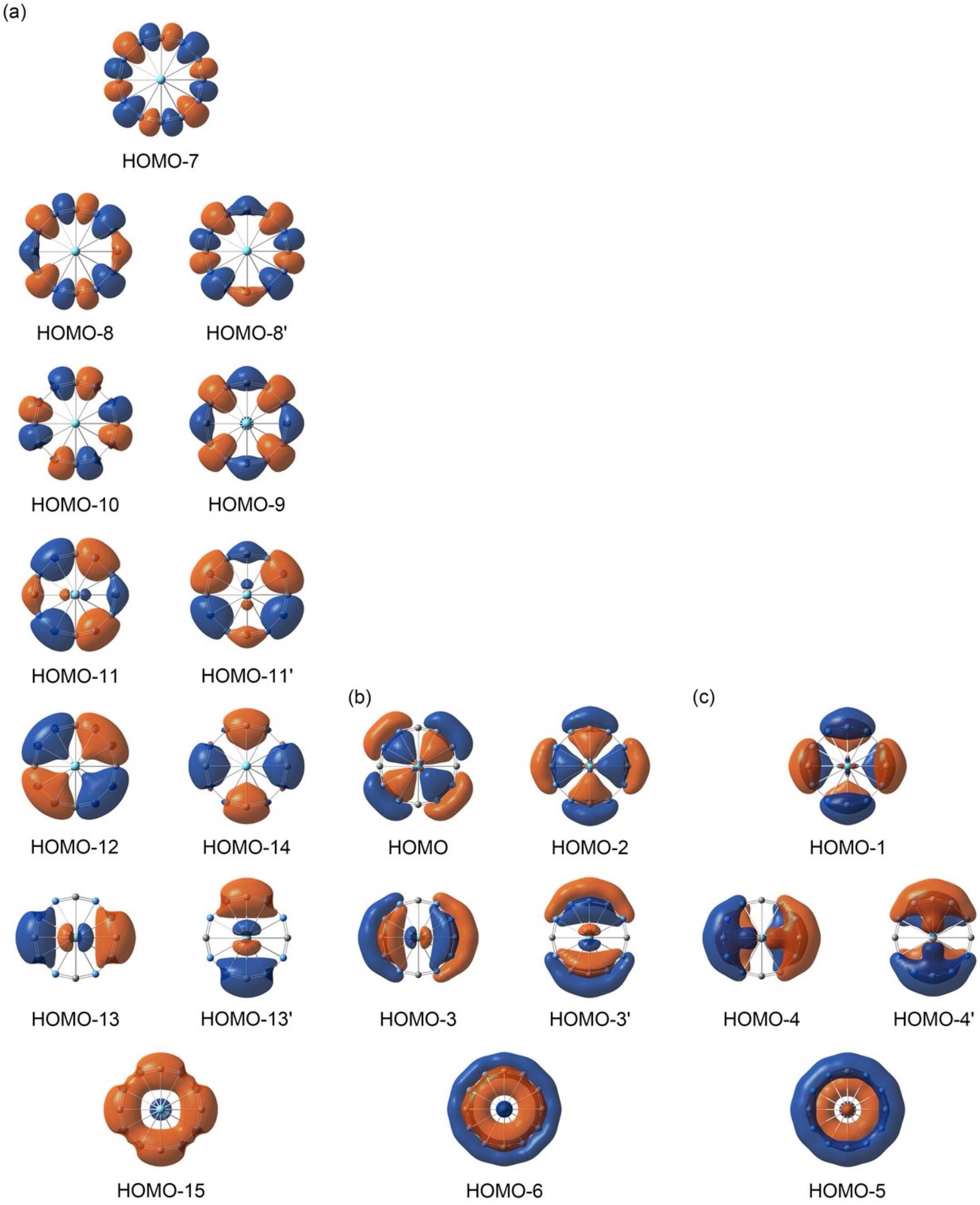

Figure S7. Occupied canonical molecular orbitals (CMOs) of the D_{4h} ($^1A_{1g}$) LM La©$B_8C_4^-$ cluster. (a) Twelve σ CMOs for 2c-2e B−C/B−B Lewis σ bonds in B_8C_4 ring. (b) Five delocalized σ CMOs in La©$B_8C_4^-$. (c) Five delocalized π CMOs in La©$B_8C_4^-$.

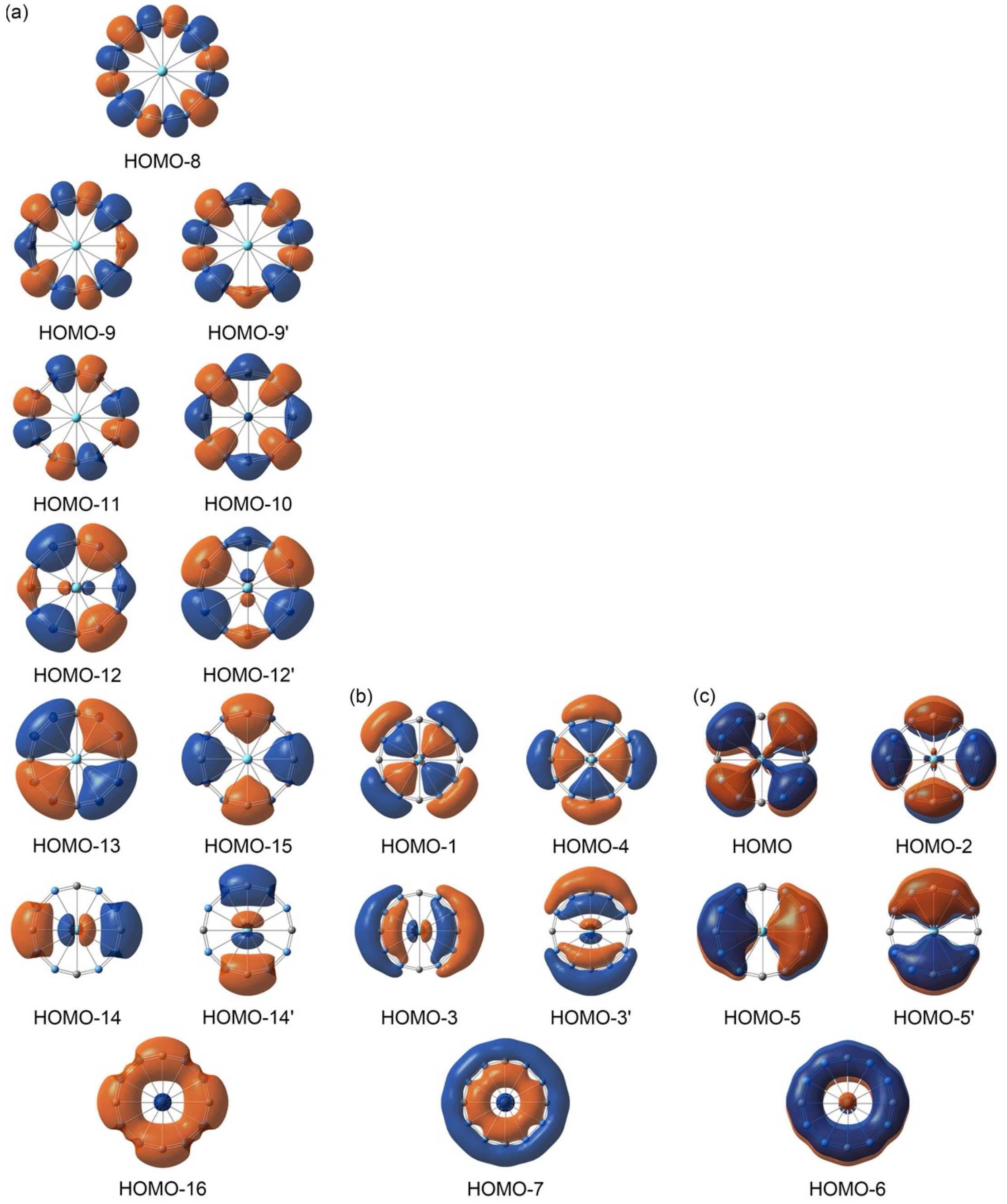

Figure S8. Occupied canonical molecular orbitals (CMOs) of the C_{4v} ($^2B_1$) GM La©B_8C_4 cluster. (a) Twelve σ CMOs for 2c-2e B−C/B−B Lewis σ bonds in B_8C_4 ring. (b) Five delocalized σ CMOs in La©B_8C_4. (c) Five delocalized π CMOs in La©B_8C_4, the SOMO represents single occupation.

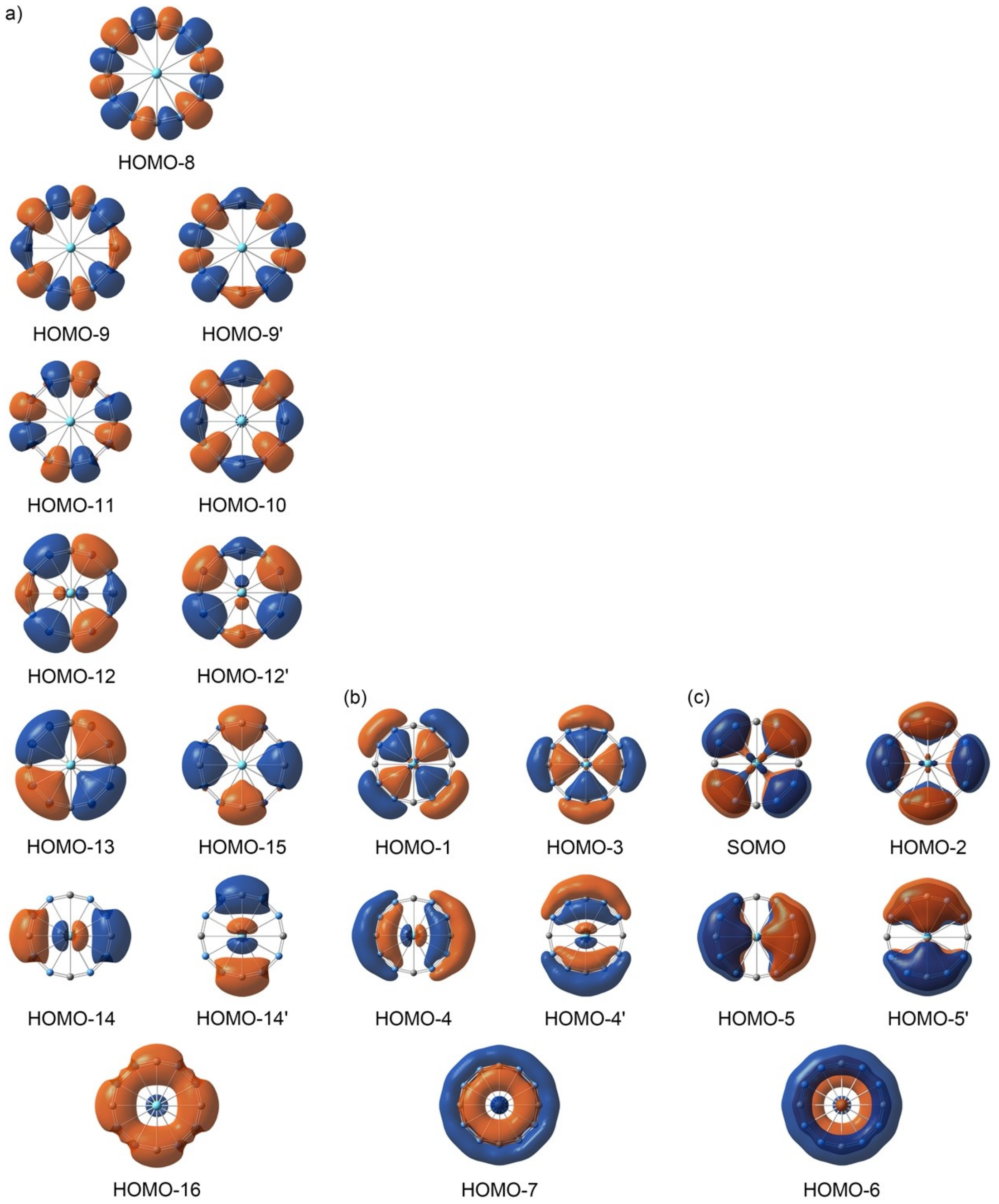

Figure S9. Chemical bonding pattern of the C_{4v} ($^2B_1$) GM La©B_8C_4 cluster based on the unrestricted adaptive natural density partitioning (UAdNDP) analysis. Occupation numbers (ONs) are indicated.

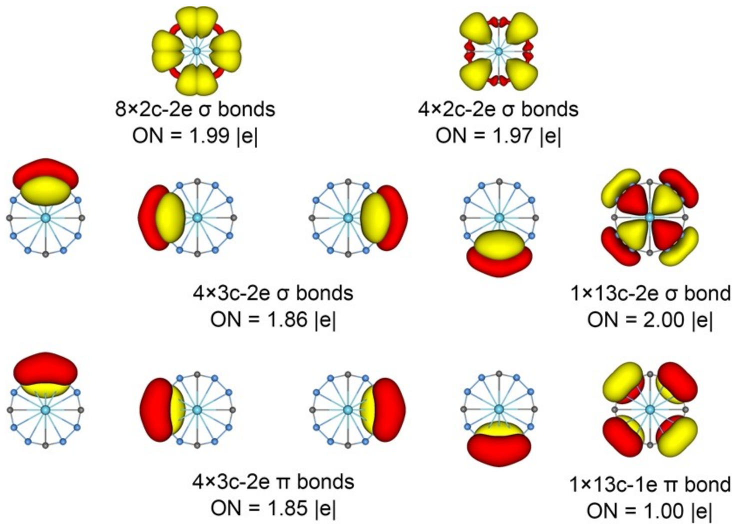

Figure S10. The calculated σ and π-ring current images of C_{4v} ($^1A_1$) GM La©$B_8C_4^+$ and D_{4h} ($^1A_{1g}$) LM La©$B_8C_4^-$ clusters. The external magnetic field is perpendicular to the molecular wheels.

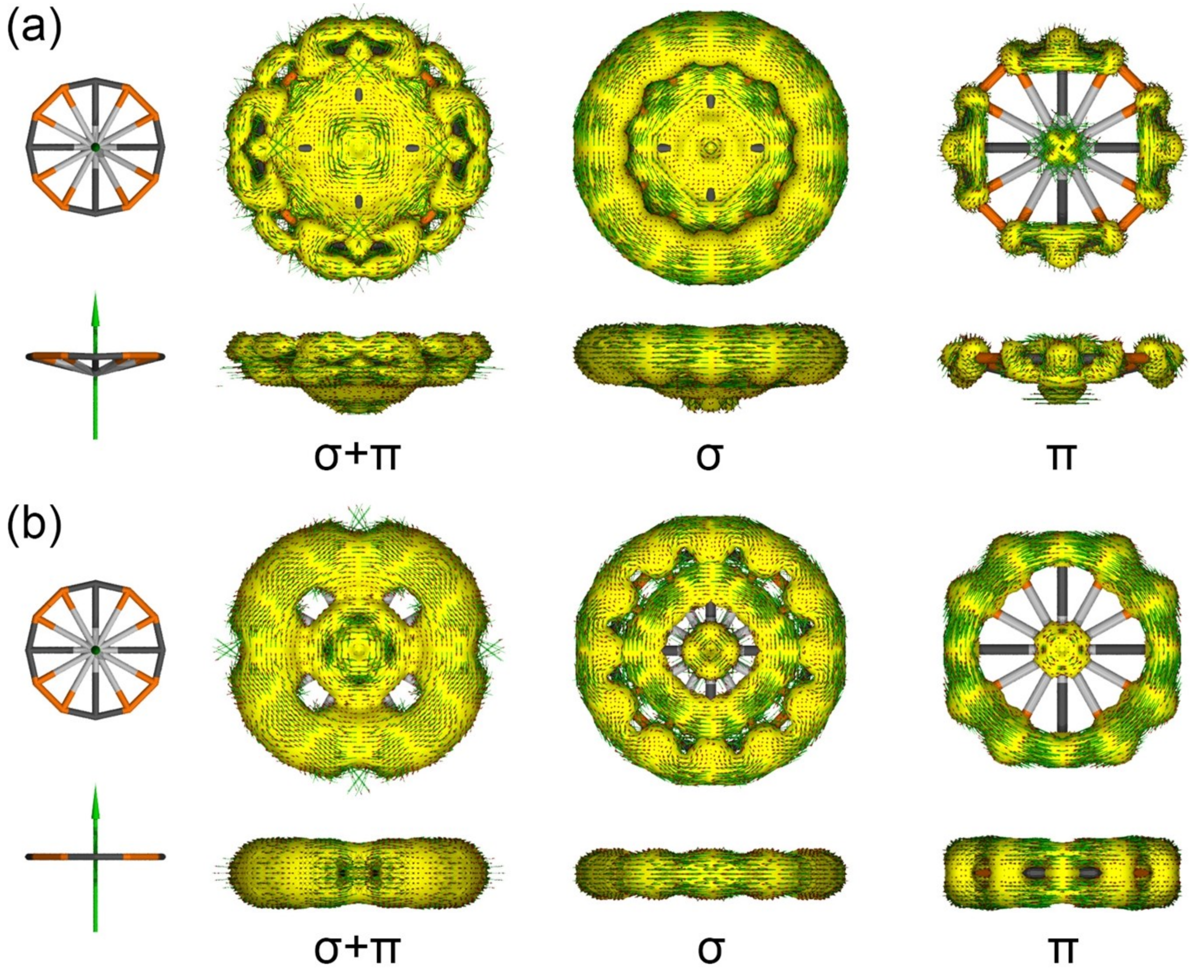

Figure S11. The energy cycle of C_{4v} ($^2B_1$) GM La©B_8C_4 cluster, along with the isomerization energy of B_8C_4 ring, bond dissociation energy (BDE) and inherent interaction energy between the central Y and B_8C_4 ring (in kcal mol^{-1}).

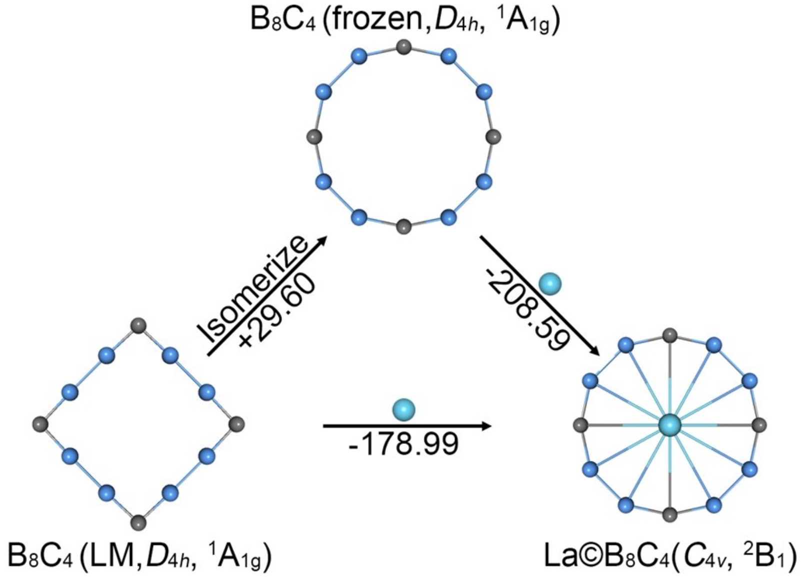

Table S1. Cartesian coordinates for the C_{4v} ($^1A_1$) GM $La©B_8C_4^+$, C_{4v} ($^2B_1$) GM $La©B_8C_4$, and D_{4h} ($^1A_{1g}$) LM $La©B_8C_4^-$ clusters at B3LYP level.

$La©B_8C_4^+$ GM (C_{4v}, $^1A_1$)

B	1.35113700	2.46442600	-0.36429900
B	-1.35113700	2.46442600	-0.36429900
B	-2.46442600	1.35113700	-0.36429900
B	-2.46442600	-1.35113700	-0.36429900
B	-1.35113700	-2.46442600	-0.36429900
B	1.35113700	-2.46442600	-0.36429900
B	2.46442600	-1.35113700	-0.36429900
B	2.46442600	1.35113700	-0.36429900
C	0.00000000	2.75120900	-0.30958500
C	2.75120900	0.00000000	-0.30958500
C	0.00000000	-2.75120900	-0.30958500
C	-2.75120900	0.00000000	-0.30958500
La	0.00000000	0.00000000	0.38600000

$La©B_8C_4$ GM (C_{4v}, $^2B_1$)

B	1.35917900	2.46062800	-0.22652300
B	-1.35917900	2.46062800	-0.22652300
B	-2.46062800	1.35917900	-0.22652300
B	-2.46062800	-1.35917900	-0.22652300
B	-1.35917900	-2.46062800	-0.22652300
B	1.35917900	-2.46062800	-0.22652300
B	2.46062800	-1.35917900	-0.22652300

B	2.46062800	1.35917900	-0.22652300
C	0.00000000	2.75395800	-0.20845200
C	2.75395800	0.00000000	-0.20845200
C	0.00000000	-2.75395800	-0.20845200
C	-2.75395800	0.00000000	-0.20845200
La	0.00000000	0.00000000	0.24673200

La©$B_8C_4^-$ LM (D_{4h}, $^1A_{1g}$)

B	1.36568600	2.45956300	0.00000000
B	-1.36568600	2.45956300	0.00000000
B	-2.45956300	1.36568600	0.00000000
B	-2.45956300	-1.36568600	0.00000000
B	-1.36568600	-2.45956300	0.00000000
B	1.36568600	-2.45956300	0.00000000
B	2.45956300	-1.36568600	0.00000000
B	2.45956300	1.36568600	0.00000000
C	0.00000000	2.76590000	0.00000000
C	2.76590000	0.00000000	0.00000000
C	0.00000000	-2.76590000	0.00000000
C	-2.76590000	0.00000000	0.00000000
La	0.00000000	0.00000000	0.00000000
